# Focus Groups Move Online: Feasibility of Tumblr Use for eHealth Curriculum Development

**DOI:** 10.2196/resprot.3432

**Published:** 2015-03-27

**Authors:** Diane Elliot, Diane Rohlman, Megan Parish

**Affiliations:** ^1^Oregon Health & Science UniversityDepartment of MedicinePortland, ORUnited States; ^2^Oregon Health & Science UniversityOregon Institute of Occupational Health SciencesPortland, ORUnited States; ^3^The University of IowaDepartment of Occupational and Environmental HealthIowa City, IAUnited States

**Keywords:** Tumblr, focus group, crowdsourcing, curriculum development, Internet

## Abstract

**Background:**

Constructing successful online programs requires engaging potential users in development. However, assembling focus groups can be costly and time consuming.

**Objective:**

The aim of this study is to assess whether Tumblr can be used to prioritize activities for an online younger worker risk reduction and health promotion program.

**Methods:**

Younger summer parks and recreation employees were encouraged to visit Tumblr using weekly announcements and competitions. Each week, new activities were posted on Tumblr with linked survey questions. Responses were downloaded and analyzed.

**Results:**

An average of 36 young workers rated each activity on its likeability and perceived educational value. The method was feasible, efficient, and sustainable across the summer weeks. Ratings indicated significant differences in likeability among activities (*P*<.005).

**Conclusions:**

Tumblr is a means to crowdsource formative feedback on potential curricular components when assembling an online intervention. This paper describes its initial use as well as suggestions for future refinements.

## Introduction

Online health promotion programs have been successful in improving understanding and altering behaviors of adults [1-3] and adolescents [4,5]. Previously, Web-based behavior-change technology had not been applied to enhancing the safety and health promotion of younger worker safety. Teenagers and younger adults, aged 14 to 24 years, account for 13.0% (18.1 million/140.2 million) of the US labor force, and their injury rate is approximately twice that of older workers [6,7]. The majority receive little or no training as to safety and their rights as workers [8]. The National Institute for Occupational Safety and Health (NIOSH)-developed training for this worker group, Youth @ Work: Talking Safety [9], is formatted as a classroom-based curriculum. The PUSH (Promoting U through Safety & Health) project is a NIOSH-funded efficacy trial to move that content online and incorporate a wellness component to extend its reach and achieve the dimensions of Total Worker Health [10].

When online programs are used with tech-savvy teenagers and young adults, the ability to incorporate graphics and interactivity may be particularly important [11,12]. Successful technology-enabled health promotion programs have engaged their prospective participants in development, using focus groups, interviews, and advisory panels [13,14], aspects especially essential for younger users [15-17]. However, assembling convenience samples of young potential users can be challenging, costly, and time consuming [18].

Tumblr is a free, easily modifiable feature-rich platform that offers features of popular social networking and blogging sites, with easy posting of content, including images and videos. We asked whether Tumblr could be used to present and prioritize components for a younger worker online health protection and promotion program. We present the identified advantages, challenges, and potential future refinements of this technique.

## Methods

### Participants

Younger workers employed as staff in the aquatics division of a large city parks and recreation program were recruited during the summer of 2012. A letter describing the PUSH study was provided during staff orientation, and all pool workers were offered an opportunity to provide anonymous online feedback concerning different e-learning activities (brief videos, games, written content, and quizzes), with a new Tumblr activity featured each week. At weekly briefings during their employment period, pool staff supervisors reminded potential participants about Tumblr activities by providing business cards with the website address and prompting workers by offering a healthy food reward each week to the pool with the highest percent participation.

### Tumblr Use

Three research assistants explored the Internet to identify brief interactive e-learning activities relevant to worker safety, communication, and healthy lifestyles, such as videos, interactive quizzes, and online games. Potential sites were reviewed and selected to provide a variety of potentially useful formats. Research assistants tracked the time required to select and post Tumblr activities.

**Figure 1:**
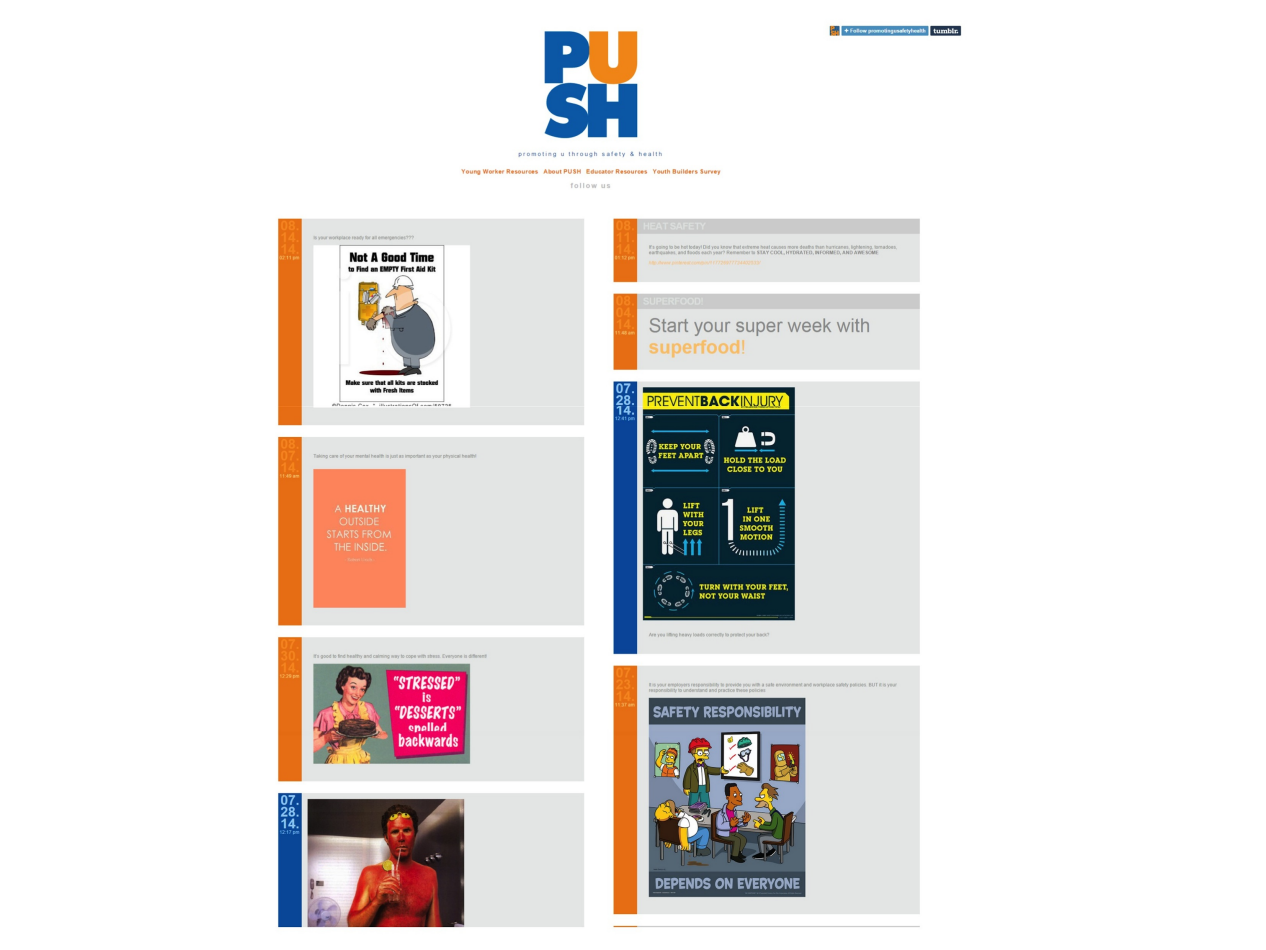
PUSH Tumblr site with different activities, one of which would be highlighted for assessment each week.

**Figure 2:**
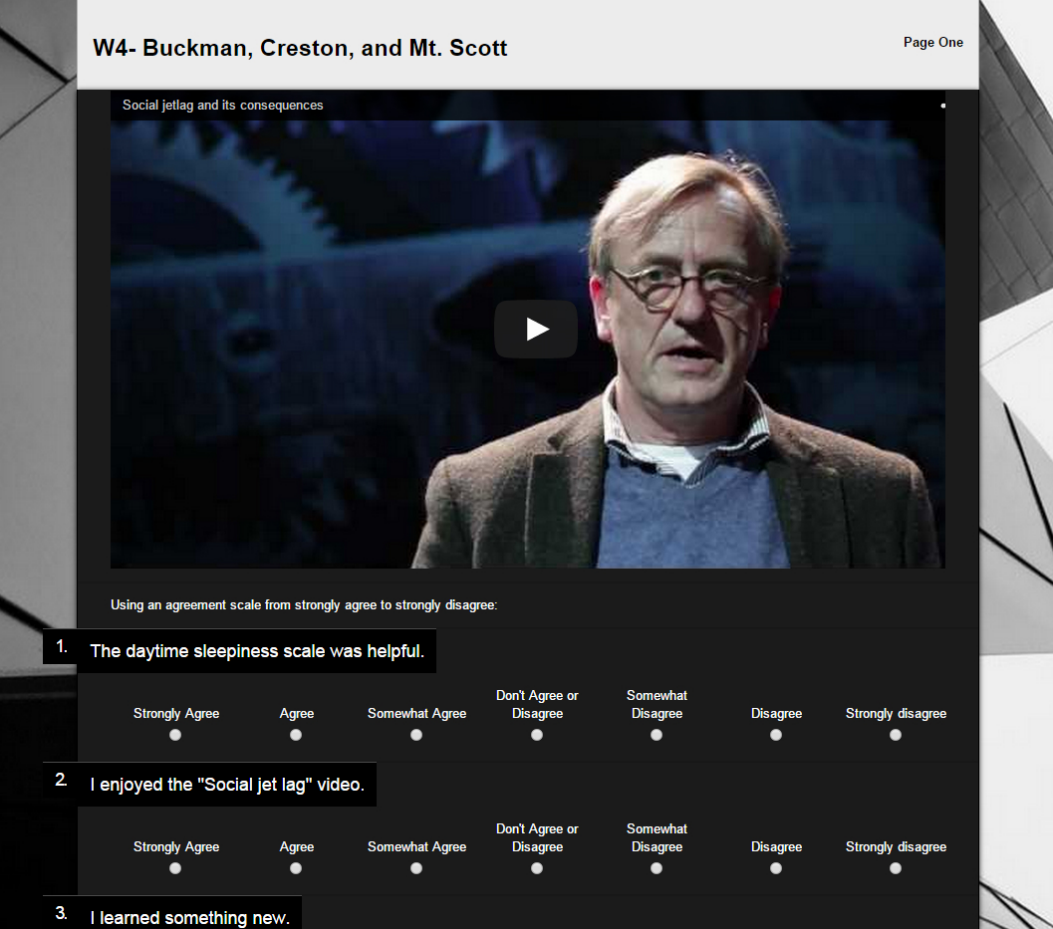
Example of Social Jet Lag video for viewing on Tumblr, with the attached segment from the linked survey that younger workers used to assess the activity.

Once chosen, activities were embedded on the PUSH project’s Tumblr page (Figures 1 and 2). Beneath the activity were open survey items powered by Survey Gizmo, an online survey hosting website. The activity was followed by instructions that survey items were for parks and recreation workers and should only be completed once per individual. The two survey questions were (1) I enjoyed the activity, (2) I learned something new. Both questions were rated on a seven-point agreement scale, from 1 = strongly disagree to 7 = strongly agree. Other than their assigned pool, responses were anonymous.

### Analysis

Data for each activity were exported to SPSS, Version 21. Descriptive statistics were compiled, and ratings among activities were compared with ANOVA.

This project was a sub-study of a larger assessment that involved an additional confidential online survey completed by participants [19]. The overarching objective was to develop content that would enhance development of the PUSH online curriculum, a Web-based worker safety and health promotion program for younger workers. The Oregon Health & Science University Institutional Review Board approved the study materials and procedures.

## Results

### Participant Demographics and Technology Use

Tumblr assessments were a component of a larger project to identify the current habits and educational needs of younger workers, and those findings have been reported [19]. The participants were a convenient sub-sample of that study. For the entire group, the mean age was 17.9 (SD 2.3), 87.2% (163/187) were white, and 65.2% (122/167) were female. As a group, 96.8% (181/187) used the Internet daily, 73.8% (138/187) checked Facebook each day, and 13.9% (26/187) visited Tumbler daily.

### Activities Assembly and Likability and Learning Ratings

The seven assessed activities are shown in Textbox 1 in the order that they were presented across the summer weeks. The three research assistants estimated each allowed 60 minutes per week to search for potential sites, and another 20 minutes comparing their best finds to decide on that week’s activity. Thirty minutes was needed to post the activity and survey questions, and another 30 minutes to download and analyze that week’s results. Pool recreation workers were reminded about that week’s activity and contest during their usual Monday supervisor briefing. Another two hours per week was needed to determine pool participation and provide the healthy food offering to the winning pool. The total time needed each week was approximately 6.5 hours.

**The seven assessed activities.**

Assessed activities

- Communication style self-assessment quiz
- Violence: news story about workplace shooting
- Self-assessment quiz about definition and types of harassment
- Speaking up: animated video about worker rights, responsibility, and self-advocacy
- Stress comical video
- Brief documentary-style video about jet lag and sleep debt
- Animated comical video highlighting workplace injuries

**Figure 3:**
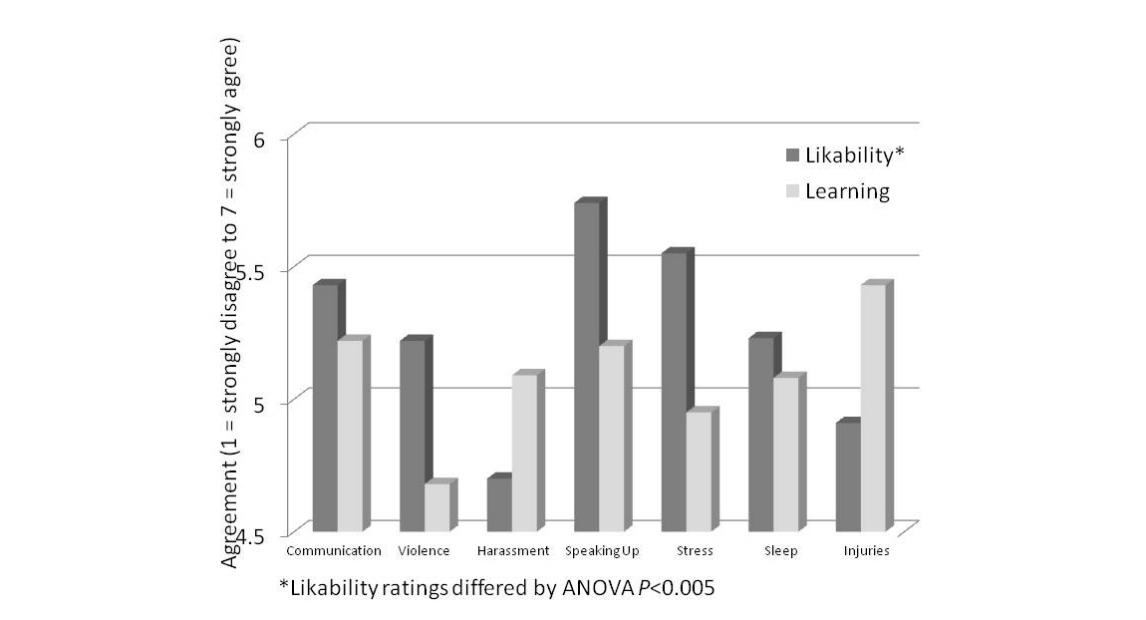
Likeability and learning ratings for Tumblr posted activities.

A mean of number 36.1 (SD 17.6) assessments (range 22-63) were received for each of the seven activities. The mean percentage of workers present at the weekly briefings was 30.0% (SD 18), and the range was wide at 7.0-71.0% across the 13 pools. Having access to a computer at the pool appeared to facilitate participation. For the activities, mean likability and learning varied, and the two scores did not parallel each other (Table 2 and Figure 3). Although there was no significant difference among the perceived learning (*F*_6,251_=0.96, *P*=.45), likability differed among activities (F_6,246_=3.35, *P*<.005).

**Table 2:** Mean likeability and perceived learning.

Activity	Likability^a^	Learning
	mean (SD)	mean (SD)
Communication	5.43 (1.04)	5.22 (1.57)
Violence	5.22 (1.48)	4.68 (1.82)
Harassment	4.70 (1.52)	5.09 (1.26)
Speaking Up	5.74 (0.97)	5.20 (1.03)
Stress	5.55 (1.23)	4.95 (1.63)
Sleep	5.23 (1.42)	5.08 (1.78)
Injuries	4.9 (1.72)	5.43 (1.16)

^a^Differences among ratings by ANOVA *P*<.005

## Discussion

### Principal Results

This is the first description of using Tumblr and a linked survey to obtain formative information for online curriculum development. The method was feasible and easily implemented with limited resources. Feedback was acquired using a method that simulated how the activities ultimately would be used in an online Total Worker Health enhancement program for younger workers.

Our planned intervention platform is a cTRAIN system that uses self-paced informational screens and periodic learning self-assessments [20]. Although the system has been shown to increase knowledge, a concern was that its relatively static images would not engage younger workers to complete the program. Tumblr was used to identify more dynamic and engaging activities to punctuate the curriculum. Ratings for likability differed significantly among posted activities, which allowed prioritizing their use in the online worker health protection and promotion program.

Using this method required access to a group with computer access that was motivated to repeatedly visit the site and comparable to those targeted in the intervention. The survey responses allowed activities to be ranked and identified significant differences in likability. The time required to assemble, post, and analyze activities was much less than would have been needed to recruit, conduct, and analyze group interviews with this number of respondents. Three refinements would enhance this method’s future use. Response rate was relatively low, and individual incentives, used in other aspects of this project, may have been more effective than the pool-wide competitions. In addition, although ratings allowed for sites to be prioritized, presenting more than one activity simultaneously and asking participants to rank rather than individually score offerings may have been more discriminatory. Finally, although inexpensive and efficient, findings lacked the richness of a facilitated discussion and adding a blog feature and chat wall might have provided more formative data. However, our goal was to select among existing components rather develop new content, and for that purpose, this method was acceptable.

### Limitations

This novel use of Tumblr for curriculum development has limitations. Our sample was younger than older adults who may visit the Web less often, and this process may not be as applicable with other less technology savvy groups. In addition, the process worked well for selecting among activities rather than designing formats. For the latter, the ability to query focus group participants would be useful. However, Tumblr use was an efficient means to obtain feedback from a range of participants without the time and expense needed to convene, conduct and analyze multiple focus groups.

### Conclusion

Tumblr use might be considered a type of crowdsourcing, a term used to describe when online tasks are performed by a network of people responding to an open call [21]. Initially used for computer-coding tasks, it has expanded to product development and marketing research [22]. Data gathered in these ways are comparable to more traditional paradigms, while being cheaper, easier and faster [23-25]. As interventions are developed and moved online, using the same vehicle to virtually focus group input is a logical extension. These novel finding provide a guide to that process.

## Abbreviations

NIOSH: National Institute for Occupational Safety and Health
PUSH: Promoting U through Safety & Health
